# Supplementary material for: Predictors of Perceived Posttraumatic Growth and Depreciation as Outcomes of Experienced Discrimination
Source: Behav Sci (Basel). 2025 Dec 24;16(1):41. doi: 10.3390/bs16010041 (PMC12838022; doi:10.3390/bs16010041)
Supplement: Supplementary file 1 [file behavsci-16-00041-s001.zip › behavsci-4004031-supplementary.pdf]

Supplemental Table S1. Correlations among all variables (Racial Discrimination Subsample).

Variable	M(SD)	2	3	4	5	6	7	8	9	10	11
1. PPTG	47.19 (22.79)	.32***	.44***	.37***	.36***	.51***	.26**	.14^{ns}	.10^{ns}	.09^{ns}	.10^{ns}
2. PPTD	24.53 (19.98)	–	.42***	.32***	.38***	.45***	.06^{ns}	.17*	.24**	.28***	.26**
3. PTSS	41.07 (15.69)		–	.68***	.70***	.60***	-.07^{ns}	.29***	.44***	.45***	.40***
4. Event Centrality	16.91 (7.02)			–	.68***	.69***	-.04^{ns}	.23**	.44***	.40***	.45***
5. Perceived Injustice	15.04 (10.22)				–	.69***	-.02^{ns}	.18*	.41***	.38***	.41***
6. Core Beliefs	16.37 (10.67)					–	.07^{ns}	.21**	.42***	.42***	.46***
7. Resilience	64.35 (18.45)						–	-.09^{ns}	-.21**	-.19*	-.27***
8. Insomnia	9.72 (5.73)							–	.34***	.37***	.36***
9. Depression	4.15 (3.65)								–	.86***	.87***
10. Anxiety	4.74 (3.75)									–	.82***
11. Stress	5.47 (4.52)										–

* $p < .05$, ** $p < .01$, *** $p < .001$, *ns* = not significant ($p > .05$). PPTG = Perceived Posttraumatic Growth. PPTD = Perceived Posttraumatic Depreciation. PTSS = PTSD Symptoms.

Supplemental Table S2. Correlations among all variables (Gender Discrimination Subsample).

Variable	M(SD)	2	3	4	5	6	7	8	9	10	11
1. PPTG	40.02 (20.43)	.29*	.38**	.46***	.57***	.64***	.10^{ns}	.03^{ns}	.28*	.32*	.33*
2. PPTD	22.29 (16.80)	–	.59***	.53***	.48***	.50***	-.36**	.32*	.51***	.48***	.45***
3. PTSS	45.80 (18.05)		–	.75***	.70***	.72***	-.37**	.45***	.66***	.53***	.71***
4. Event Centrality	17.85 (5.81)			–	.80***	.79***	-.19^{ns}	.22^{ns}	.45***	.33*	.48***
5. Perceived Injustice	17.41 (11.39)				–	.76***	-.26^{ns}	.41**	.51***	.44**	.55***
6. Core Beliefs	19.32 (10.76)					–	-.17^{ns}	.24^{ns}	.46***	.51***	.57***
7. Resilience	61.25 (16.85)						–	-.46***	-.54***	-.41**	-.46***
8. Insomnia	10.94 (6.84)							–	.56***	.50***	.63***
9. Depression	5.31 (3.46)								–	.84***	.84***
10. Anxiety	5.92 (3.69)									–	.79***
11. Stress	6.56 (4.14)										–

* p < .05, ** p < .01, *** p < .001, *ns* = not significant (p > .05). PPTG = Perceived Posttraumatic Growth. PPTD = Perceived Posttraumatic Depreciation. PTSS = PTSD Symptoms.
